# Structured Promoter Variability in Epigenetically Regulated Operons Contributes to Surface Adaptation in *Salmonella*

**DOI:** 10.1111/1751-7915.70312

**Published:** 2026-02-12

**Authors:** Rocío Fernández‐Fernández, Gabriel Gutiérrez, Francine Piubeli, Junkal Garmendia, Carmen R. Beuzón, María Antonia Sánchez‐Romero

**Affiliations:** ^1^ Departamento de Microbiología y Parasitología, Facultad de Farmacia Universidad de Sevilla Sevilla Spain; ^2^ Departamento de Genética, Facultad de Biología Universidad de Sevilla Sevilla Spain; ^3^ Instituto de Agrobiotecnología, Consejo Superior de Investigaciones Científicas (IdAB‐CSIC)‐Gobierno de Navarra Mutilva Spain; ^4^ Centro de Investigación Biomédica en Red de Enfermedades Respiratorias (CIBERES) Madrid Spain; ^5^ Conexión Resistencia Antimicrobianos CSIC (AMR‐CSIC) Madrid Spain; ^6^ Departamento Biología Celular, Genética y Fisiología Instituto de Hortofruticultura Subtropical y Mediterránea 'La Mayora', Universidad de Málaga‐Consejo Superior de Investigaciones Científicas (IHSM‐UMA‐CSIC) Málaga Spain

## Abstract

Bacterial adaptation to dynamic and hostile environments often relies on the ability to modify surface structures, which are subjected to strong selective pressures. While mutations in coding sequences that alter surface structures have long been recognised as key drivers of bacterial adaptation, this study reveals structured promoter variation as a novel additional layer of functional adaptation. Focusing on the *opvAB* operon in *Salmonella*, epigenetically regulated by the coordinated action of Dam methylation and OxyR binding, we investigated the evolutionary dynamics and regulatory architecture of its regulatory region. Comparative genomics of *Salmonella* subspecies revealed striking variability in this regulatory region. Notably, the *opvAB* promoter exhibited mutations clustering at specific, recurrent positions, suggesting lineage‐specific fine‐tuning through structured and potentially functional variability, further demonstrated experimentally by phase variation analysis within clonal populations at the single bacterial cell level. Our findings highlight a potentially overlooked evolutionary mechanism by which bacteria can modulate gene expression through a balance between conservation and variability in regulatory elements. This structured promoter variation likely complements genetic and epigenetic strategies, offering a new perspective on how bacterial populations adapt to environmental challenges.

## Graphical abstract

We discovered that *Salmonella* uses structured, recurrent variation in epigenetically regulated promoter regions to fine‐tune gene expression, revealing a novel evolutionary strategy for surface adaptation.

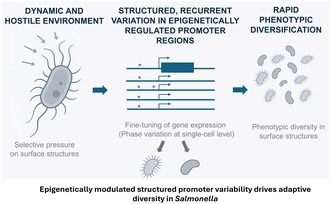

## 1 Introduction

Bacterial adaptation to diverse and often unpredictable environments is crucial for their survival and proliferation. Bacterial surface proteins play essential roles in bacterial interactions with the environment, including cell–cell interactions, ion and nutrient transport, cell signalling or antibiotic resistance; they are also important for host cell infection, defence against host responses and induction of toxicity (Niemann et al. 2004; Lebeer et al. 2010; Schneewind and Missiakas 2012; Chagnot et al. 2013; Cota, Bunk, et al. 2015; Cota, Sánchez‐Romero, et al. 2015).

Gram‐negative bacteria utilise a range of mechanisms to attach to host cells, invade tissues and survive in hostile environments. Outer membrane surface‐exposed proteins often play critical roles in the initial interaction with host cells, facilitating colonisation and infection (Silhavy et al. 2010). The outer membrane, rich in lipopolysaccharide (LPS), serves as a barrier to toxic compounds, including many antibiotics (Nikaido 2009). In addition, efflux pumps actively expel harmful substances from the cell, contributing to (multi)drug resistance (Nikaido 2009). Adhesion structures such as pili and fimbriae are essential for bacterial adherence to host tissues, an early and crucial step in pathogenesis and colonisation (Thanassi et al. 2012). Moreover, specialised secretion systems inject bacterial effectors directly into host cells to manipulate their function in favour of the pathogen (Thanassi et al. 2012). Finally, antigenic variation enables bacteria to alter their surface proteins and evade detection by the host immune system (van der Woude and Bäumler 2004).

Traditionally, genetic mutations leading to altered surface structures have been considered the primary drivers of long‐term adaptation (Bikard and Marraffini 2012). However, the capacity of bacterial populations to rapidly respond to changing selective pressures often requires more flexible mechanisms than those provided by genetic mutation (Hernández et al. 2012; Sánchez‐Romero and Casadesús 2014, 2018, 2021; Cota, Bunk, et al. 2015; Cota, Sánchez‐Romero, et al. 2015; Rodríguez Olivenza et al. 2019; Fernández‐Fernández et al. 2024). A key strategy to achieve this adaptability is the generation of phenotypic heterogeneity within clonally identical populations (Casadesús and Low 2006; Sánchez‐Romero and Casadesús 2014, 2020). This variability can be generated through various mechanisms, including epigenetic switches that result in heritable but reversible changes in gene expression without altering the underlying DNA sequence. Such epigenetic variation allows a bacterial population to explore different phenotypic states, shifting towards increased survival of a subpopulation to adverse selective conditions to ensure species persistence (Kussell and Leibler 2005; Kussell et al. 2005; Casadesús and Low 2013; Seib et al. 2020).

The ability to rapidly and reversibly modify surface structures provides a dynamic interface with the environment, potentially allowing bacteria to evade host defenses or resist phage infection without incurring the costs associated with permanent genetic alterations. This dynamic modulation can be seen as an alternative or complementary evolutionary adaptive strategy, offering a faster and more flexible route to survival compared with solely relying on the accumulation of beneficial mutations (Fernández‐Fernández et al. 2024).

Among variable phenotypic traits, bacterial surface LPS and its O‐antigen play a fundamental role in interactions with the environment, being crucial for successful infection. The LPS comprises three structural domains covalently linked: the lipid A, a short core oligosaccharide and the O‐antigen polysaccharide (Liu et al. 2014). In general, the lipid A and the short core oligosaccharide are highly conserved, likely reflecting their essential roles in the maintenance of the outer membrane integrity (Heinrichs et al. 1998). On the other hand, the O‐antigen is considered one of the most variable cell components, and its diversity is the basis for *Salmonella*—and other pathogens—serotyping (Li et al. 2011). In *Salmonella*, LPS is important for swarming motility, intestinal colonisation, serum resistance, intracellular replication, phage resistance and resistance to killing by macrophages (Kong et al. 2011). Moreover, LPS structure can alter the presence and the activity of virulence factors (Wandersman and Létoffé 1993; Stone and Miller 1995).

In *Salmonella enterica* , the length of the LPS O‐antigen chain undergoes complex regulation that includes the *opvAB* operon (Cota et al. 2012). This phase‐variable locus encodes two inner membrane proteins that modify the O‐antigen chain's length. The *opvAB* phase variation is controlled by an epigenetic mechanism involving both methylation of GATC sites in its regulatory region by the Dam enzyme and the action of the oxidative stress‐sensitive transcription factor OxyR. Differential binding of OxyR to the *opvAB* regulatory region, influenced by the DNA methylation state, is crucial for the formation of OpvAB^OFF^ and OpvAB^ON^ cell lineages. The OpvAB^OFF^ state produces long O‐antigen chains associated with virulence, while the OpvAB^ON^ state produces shorter O‐antigen chains, which can confer resistance to certain bacteriophages (Cota et al. 2012; Cota, Bunk, et al. 2015; Cota, Sánchez‐Romero, et al. 2015). This reversible switching highlights the adaptive value of epigenetic variation in generating bacterial subpopulations with differential susceptibilities to various environmental pressures, potentially serving as a form of ‘mutation avoidance’ by providing phenotypic plasticity in the face of selective challenges (Phillips et al. 2019; Seib et al. 2020; Fernández‐Fernández et al. 2024). Exploring such epigenetic mechanisms on loci related to bacterial surface antigens offers valuable insights into the sophisticated strategies employed by bacteria to thrive in fluctuating environments.

In this study, we investigated the sequence diversity of genes associated with the bacterial surface across the *Salmonella* genus, with a particular focus on elucidating the evolutionary dynamics and regulatory architecture of the *opvAB* operon—a key horizontally acquired locus that modulates O‐antigen chain length and mediates a trade‐off between virulence and resistance to bacteriophages. Through the comparative analysis of sequences in various subspecies and strains, we investigated the conservation and diversity of the *opvAB* locus regulatory elements across the *Salmonella* genus. We complemented our in silico analysis of public genome databases with field bacterial isolates to analyse the functional consequences of variation in key regulatory sites. These isolates, obtained from poultry farms, enabled us to directly examine the impact of promoter sequence variation on gene expression and, consequently, on the potential for phase variation. The results obtained provide a detailed insight into the evolution of an epigenetic system in *Salmonella*, acquired by horizontal gene transfer and involved in bacterial adaptation to dynamic and unpredicted environments.

## 2 Results and Discussion

### 2.1 Diversity on Surface‐Associated Antigens in the Genus *Salmonella*

Bacteria interact with their environments by employing strategies that promote survival, such as generating surface variability to adapt to changing conditions and evade host immune pressures. We aimed to explore the genetic diversity of *Salmonella* surface‐associated antigens to better understand the evolutionary adaptations of bacterial surfaces involved in host interaction and pathogenesis. We selected the genus *Salmonella* as a model organism due to its extensive variability in terms of surface structures, which are key determinants of host‐pathogen interactions and survival in diverse environmental conditions (Fierer and Guiney 2001; Yue and Schifferli 2014; Bernal‐Bayard and Ramos‐Morales 2018). Specifically, we examined the coding sequences (CDSs) of two groups of genes across fully annotated *Salmonella* genomes retrieved from the NCBI database: (i) surface‐associated genes, which constituted the main focus of this study, and (ii) genes involved in the translational machinery, which were used as housekeeping genes because they encode core components of protein synthesis that are essential for cellular viability. Both groups were identified based on their Gene Ontology (GO) annotations, as detailed in the Methods section. We used BLASTn (Nucleotide Basic Local Alignment Search Tool) to calculate the percent identity (PI) of each CDS relative to a reference strain ( *S. enterica* serovar Typhimurium 14028S), selected for its well‐annotated genome and its wide use as a model strain in *Salmonella* research.

**FIGURE 1:**
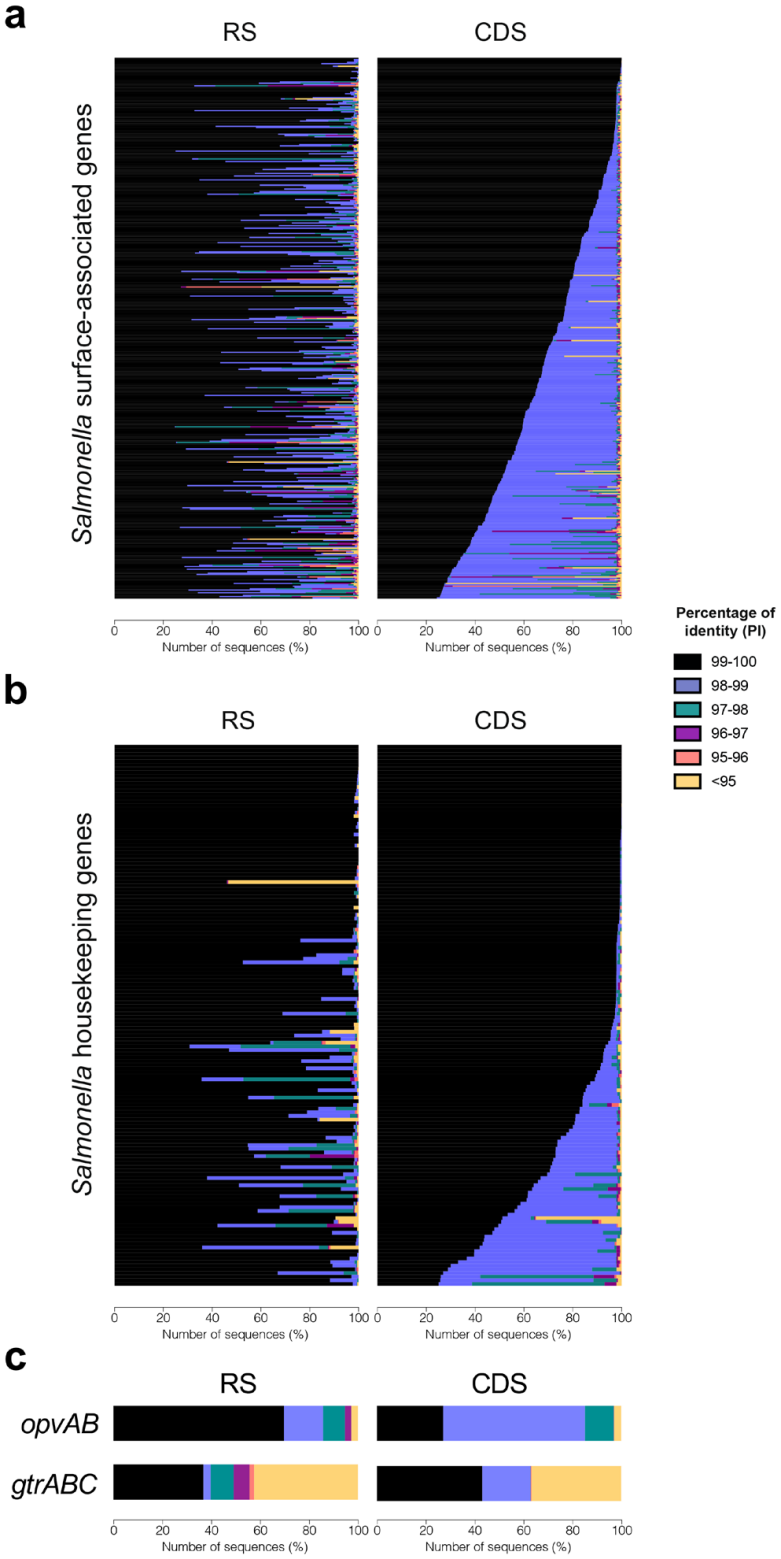
Comparative sequence diversity of functionally distinct gene categories in the *Salmonella* genus. Each row represents a *Salmonella* gene, for which nucleotide sequence diversity was assessed in both the upstream regulatory sequence (RS) and the coding sequence (CDS). Sequence diversity is represented as the proportion of sequences falling within defined percentage identity (PI) intervals relative to the reference sequence ( *S. enterica* serovar Typhimurium 14028S). PI values are grouped into 1% intervals and represented by colour coding. Genes are ranked in descending order according to the proportion of sequences within the 99%–100% PI range for the CDS. (a) Analysis of surface‐associated genes. (b) Analysis of translation‐related genes. (c) Analysis of the epigenetically regulated operons *opvAB* (encoding proteins essential for O‐antigen repeat unit polymerisation) and *gtrABC* (encoding glycosyltransferases responsible for the addition of sugar residues to the O‐antigen). Functional gene classification into surface‐associated and translation‐related categories was based on Gene Ontology (GO) Process terms, as detailed in Table S1.

Our comparative analysis revealed distinct patterns of CDS conservation across functional categories (Figure 1). Surface‐associated genes—which include loci responsible for nutrient and ion transport, cell‐envelope biogenesis (e.g., LPS and O‐antigen assembly), protein secretion systems (Types I, II and VI) and motility or adhesion (flagella and pili)—exhibited significantly higher sequence variability, characterised by a wide distribution of PI values (Figure 1a). In stark contrast, housekeeping genes involved in the translational machinery—such as those for ribosome biogenesis, tRNA aminoacylation and rRNA processing—displayed high levels of sequence conservation (Figure 1b). This divergence in conservation levels likely reflects the contrasting adaptive pressures acting on these systems: while essential intracellular processes require genomic stability, genes mediating interactions with the external environment must remain flexible to facilitate nutrient acquisition and immune evasion. These findings support an evolutionary trade‐off wherein the genome maintains high fidelity for core survival functions while permitting rapid diversification in genes that interface with dynamic and potentially hostile environments (Koonin and Wolf 2010).

While traditional views of evolution emphasise mutations within coding sequences, changes in the regulatory regions could also be a source of variability enabling bacteria to respond to environmental challenges. To explore this, we investigated the conservation of regulatory sequences—specifically, an upstream region proximal to the start codon of each gene (referred to as the ‘regulatory sequence’ or RS in Figure 1). Our objective was to analyse the correlation between the PI of the RS and the CDS to determine whether specific loci act as mutational hotspots and to assess how these regions tolerate genetic variation. By calculating the mean PI for each of the 4743 genes in the *Salmonella* genome, we generated a scatter plot and determined the Pearson correlation coefficient (*R* = 0.311, *p* < 2.22 × 10^−16^) (Figure S1). While the analysis shows a statistically significant positive relationship, meaning highly conserved coding sequences generally correspond with conserved regulatory regions, the low *R*‐value indicates that evolutionary pressures acting on these two regions are partially independent. Although CDS identity is generally higher and more consistent, we observed that in certain loci the opposite occurs: the regulatory region remains more conserved than its corresponding coding sequence. This suggests that these regions can diverge independently, indicating that mutation rates or selective pressures differ significantly between the CDS and RS at specific loci.

To explore this diversity in greater detail, we transitioned from analysing average PI to a binned interval analysis across specific functional categories (Figure 1a,b). This approach allowed us to contrast the evolutionary behaviour of cell‐surface‐associated genes against those of the translational machinery. The trend of independent evolution remained consistent but was particularly accentuated in surface‐associated loci. The lack of a direct, strong correlation in these genes suggests they evolve under a high degree of independence, a phenomenon that likely facilitates rapid adaptation to external pressures, such as host immune responses.

Moving beyond general genomic trends, we sought to understand how genomic sequences evolve and accumulate changes specifically within loci subject to epigenetic regulation. To investigate this, we focused on two well‐characterised, epigenetically regulated operons involved in the synthesis of the LPS O‐antigen: *opvAB* and *gtrABC* (Broadbent et al. 2010; Cota et al. 2012; Cota, Bunk, et al. 2015; Cota, Sánchez‐Romero, et al. 2015). As these are cell‐surface genes, these operons serve as primary models for examining how the requirement for epigenetic control influences mutational patterns and sequence divergence in regions exposed to intense selective pressure. As shown in Figure 1c, the regulatory regions of the *opvAB* and *gtrABC* operons exhibit notably low conservation across genomes. Both operons are regulated by phase variation mechanisms mediated by Dam methylation and the transcriptional regulator OxyR, which facilitates reversible switching between ‘ON’ and ‘OFF’ expression states. This stochastic switching alters the bacterial surface through O‐antigen modification, generating phenotypic heterogeneity that enhances *Salmonella*'s fitness in fluctuating environments (Broadbent et al. 2010; Cota et al. 2012; Cota, Bunk, et al. 2015; Cota, Sánchez‐Romero, et al. 2015; Fernández‐Fernández et al. 2024). The low sequence identity found in these promoters is particularly striking given their regulatory complexity and functional relevance. This suggests that, beyond the immediate flexibility provided by epigenetic switching, evolutionary changes in regulatory DNA regions may further contribute to fine‐tuning the expression of surface antigens, adding an additional layer of variability to already known genetic and epigenetic mechanisms.

Since the molecular mechanism and functional architecture of the *opvAB* regulatory region have already been defined (Cota, Bunk, et al. 2015; Cota, Sánchez‐Romero, et al. 2015), from this point forward, we mainly focus on the *opvAB* operon as a model locus to explore these processes in detail.

### 2.2 Diversity on the *opvAB* Epigenetic System in the Genus *Salmonella*

As previously described, the *opvAB* operon controls the O‐antigen chain length in the LPS of *Salmonella* and undergoes transcriptional switching between ON and OFF states through a process mainly governed by the transcription factor OxyR and by DNA adenine methylation which, without genetic modifications, provides a selective advantage (Cota, Bunk, et al. 2015; Cota, Sánchez‐Romero, et al. 2015). The *opvAB* operon thus offers an excellent model for investigating the complex interplay between genetic and epigenetic modifications.

**FIGURE 2:**
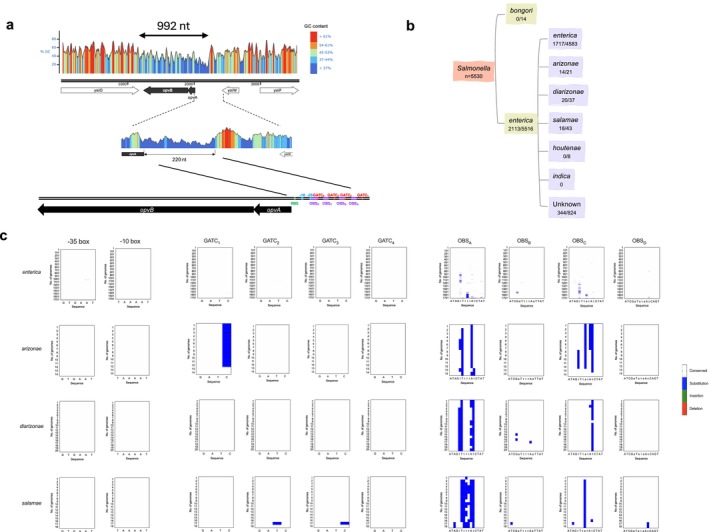
The *opvAB* operon in the genus *Salmonella*. (a) Delimitation of the *opvAB*‐containing DNA fragment acquired by horizontal gene transfer (HGT). Analysis of GC content of *opvAB* and neighbouring regions. The sequence of *Salmonella enterica* subsp. *enterica* serovar Typhimurium ATCC 14028 was analysed using SnapGene software. Diagram of GC content of the sequence located between *opvA* and *yeiW* genes. GC content suggests the boundary of the operon is located 220 nucleotides upstream of the *opvA* gene. The promoter‐proximal boundary of the HGT fragment is located 220 nucleotides upstream of *opvA* ATG. The diagram depicts the ribosome binding site, RBS (green), the −35 and −10 promoter modules (blue), the four GATCs (red) found in the upstream activating sequence (UAS) and the four OxyR binding sites, OBS_A_ OBS_B_ OBS_C_ and OBS_D_ (purple). (b) Assortment of the 992‐nucleotide sequence containing the *opvAB* operon in 5530 *Salmonella* genomes available in the NCBI database, classified by species and subspecies. Number of *opvAB* positive genomes among the sequences available at the NCBI database. A total of 824 genomes were annotated as *S. enterica* without subspecies‐level classification; among them, 344 contained the *opvAB* operon sequence. (c) Inter‐subspecies mutational landscape of the *opvAB* key regulatory elements across *Salmonella enterica* genomes classified by subspecies. Analysis includes mutations in the −35 and −10 promoter boxes, four GATC sites and four OxyR binding sites. Among the analysed genomes, 1710 correspond to subsp. *enterica*, 14 to subsp. *arizonae*, 20 to subsp. *diarizonae* and 18 to subsp. *salamae*. The reference sequence from strain ATCC 14028 is displayed below the heatmap. Each row represents a genome, and each column corresponds to a nucleotide position. OBS motifs are annotated following established consensus sequences, while invariant positions are shown in uppercase, degenerate positions (i.e., variable nucleotides) are shown in lowercase (Storz et al. 1990; Toledano et al. 1994). Nucleotide changes are represented by colours as follows: Substitutions (blue), insertions (green), deletions (red) and conserved positions (white).

**FIGURE 3:**
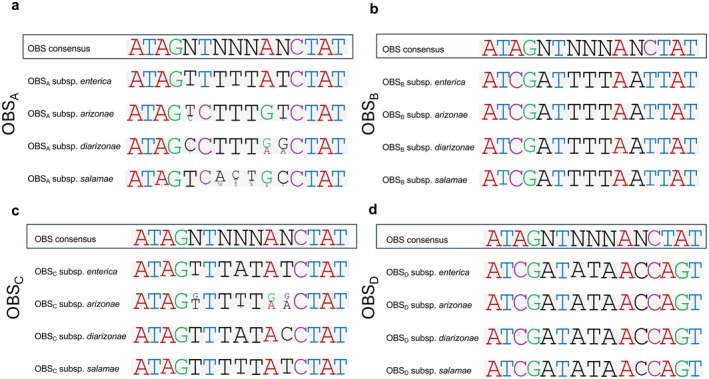
Diversity of OxyR binding sites in the *opvAB* promoter across different *Salmonella enterica* subspecies. Nucleotide sequences of the OxyR binding sites A, B, C and D within the *opvAB* promoter region (a, b, c and d, respectively). The first row shows the consensus OBS sequence as defined by (Storz et al. 1990; Toledano et al. 1994), followed by sequences from *S. enterica* subsp. *enterica*, *arizonae*, *diarizonae* and *salamae*. The size of each nucleotide reflects the relative frequency of the corresponding nucleotide across the genomes analysed. ‘N’ denotes any nucleotide. Nucleotides that are not part of the consensus sequence are shown in black, whereas consensus nucleotides are colour‐coded as follows: A in red, T in blue, G in green and C in purple.

The *opvAB* operon was previously described as potentially acquired by horizontal gene transfer based on distinctive base composition, specifically low G + C content (~38.3% in coding regions) compared with the *S. enterica* core genome (~52.2%) (Navarre et al. 2006; Cota et al. 2012). First, we used a previously delimited sequence of 992 nucleotides containing the *opvAB* operon (the putative HGT‐acquired fragment based on G + C content, Figure 2a) as a query to screen 5530 complete and fully annotated *Salmonella* genomes among strains of the genus *Salmonella* available at the NCBI database. This analysis indicates that one chromosomal copy per genome of this operon is present in *Salmonella enterica* subspecies but absent in *Salmonella bongori* and other species of enteric bacteria, suggesting a relatively recent or specific transfer event. However, given the unequal number of available genomes for these groups, this observation should be interpreted with caution and may be influenced by sampling bias. Specifically, the operon was present in 1717 of 4583 of subspecies *enterica*, in 14 of 21 subspecies *arizonae*, in 20 of 37 *diarizonae* and in 18 of 43 genomes of subspecies *salamae* (Figure 2b). Then, we conducted a detailed nucleotide sequence analysis of the promoter‐regulatory region upstream of the *opvAB* operon. The promoter‐proximal boundary of the *opvAB* fragment is located 220 nucleotides upstream of the *opvA* start codon, encompassing key regulatory elements which include the *opvAB* promoter, four GATC methylation sites and four OxyR binding sites (OBSs) (Figure 2a). Given the functional relevance of these elements in the epigenetic regulation of *opvAB* expression, sequence divergence within this region may indicate adaptive changes in gene regulation. We performed a BLASTn analysis of this 220‐nucleotide upstream region across all complete genomes of the *Salmonella* genus. The retrieved sequences were aligned to assess variation in the core regulatory motifs responsible for controlling *opvAB* transcription across different *Salmonella* subspecies (Figure 2c). From this analysis, the following conclusions were drawn:

- The −35 and −10 modules of the *opvAB* promoter were 100% conserved across all sequences of all *Salmonella* subspecies.
- GATC_1_ was conserved in 100% of sequences in subspecies *enterica*, *diarizonae* and *salamae*. GATC_1_ was not present in subspecies *arizonae*, which contains GATT instead of GATC in 13 out of 14 available sequences. A previous study in subspecies *enterica* demonstrated that the effect of GATC_1_ on the *opvAB* expression was small (Cota, Bunk, et al. 2015; Cota, Sánchez‐Romero, et al. 2015); therefore, the absence of a canonical GATC_1_ site in subspecies *arizonae* is unlikely to have a major impact on *opvAB* regulation.
- GATC_2_ was highly conserved. Out of 1762 analysed sequences, only one substitution (GATC was substituted by GAAC) was found in GATC_2_ in one subspecies *salamae* genome.
- GATC_3_ were conserved in the majority of sequences. Out of 1762 genomes analysed, only one substitution (GATC was substituted by GATT) was found in GATC_3_ in a subspecies *salamae* genome.
- GATC_4_ were fully conserved in all the 1762 sequences analysed.
- OxyR binding site A (OBS_A_) and OxyR binding site C (OBS_C_) were partially conserved, with variability mainly restricted to non‐consensus positions, and differed in subspecies *enterica*, *arizonae, diarizonae* and *salamae*. Out of the 10 consensus positions of OxyR binding sites, OBS_A_ is conserved in subspecies *enterica* (10/10), *arizonae* (8/10), *diarizonae* (9/10) and *salamae* (7/10). OBS_C_ is conserved in 10/10 positions in subspecies *enterica*, *arizonae* and *salamae*. Only in subspecies *diarizonae* is the OBS_C_ conserved in 9/10 positions (Figures 2c and 3).
- OxyR binding site B (OBS_B_) and OxyR binding site D (OBS_D_), which are embedded GATC_2_ and GATC_4_, were fully nearly conserved in all sequences analysed, even between subspecies. Out of the 10 consensus positions of OxyR binding sites, OBS_B_ and OBS_D_ are conserved in 8/10 positions in subspecies *enterica*, *arizonae*, *diarizonae* and *salamae* (Figures 2c and 3).

Despite the variability of the *opvAB* regulatory sequence being high (Figure 1c), it is noteworthy that crucial elements in the regulation of *opvAB* expression, such as −35 and −10 regions, and the four GATC motifs recognised by Dam methyltransferase, are mostly conserved (Figure 2c).

### 2.3 Intraspecific Diversity of the *opvAB* Epigenetic System in *Salmonella enterica*

**FIGURE 4:**
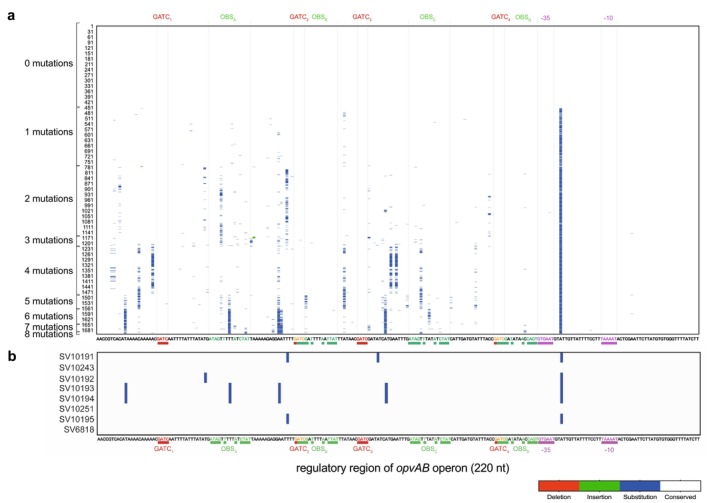
Intra‐subspecies mutational distribution in the regulatory region of the *opvAB* operon in *Salmonella enterica* subsp. *enterica*. (a) Localisation and distribution of mutations within the 220 nucleotides upstream of the *opvA* start codon, across *S. enterica* subsp. *enterica* genomes carrying between 1 and 8 mutations. Each row represents a genome, and each column corresponds to a nucleotide position. The nucleotide sequence of the ATCC 14028 strain used as reference is shown below the heatmap, with key regulatory elements such as GATC sites, OxyR binding sites (OBSs) and the −35 and −10 promoter boxes highlighted in colours. Nucleotides corresponding to conserved positions in the OBS consensus sequence are highlighted in green. Mutations are categorised as deletions (red), insertions (green) and substitutions (blue), while conserved nucleotides are represented in white. (b) Localisation and distribution of mutations within the regulatory region of *opvAB* in eight *Salmonella* strains collected in poultry farms. Each row represents one strain, and each column corresponds to a nucleotide position. The nucleotide sequence of the ATCC 14028 strain used as reference is shown below the heatmap, with key regulatory elements such as GATC sites, OxyR binding sites (OBSs) and the −35 and −10 promoter boxes highlighted in colours. Nucleotides corresponding to conserved positions in the OBS consensus sequence are highlighted in green. Mutations are categorised as deletions (red), insertions (green) and substitutions (blue), while conserved nucleotides are represented in white.

**FIGURE 5:**
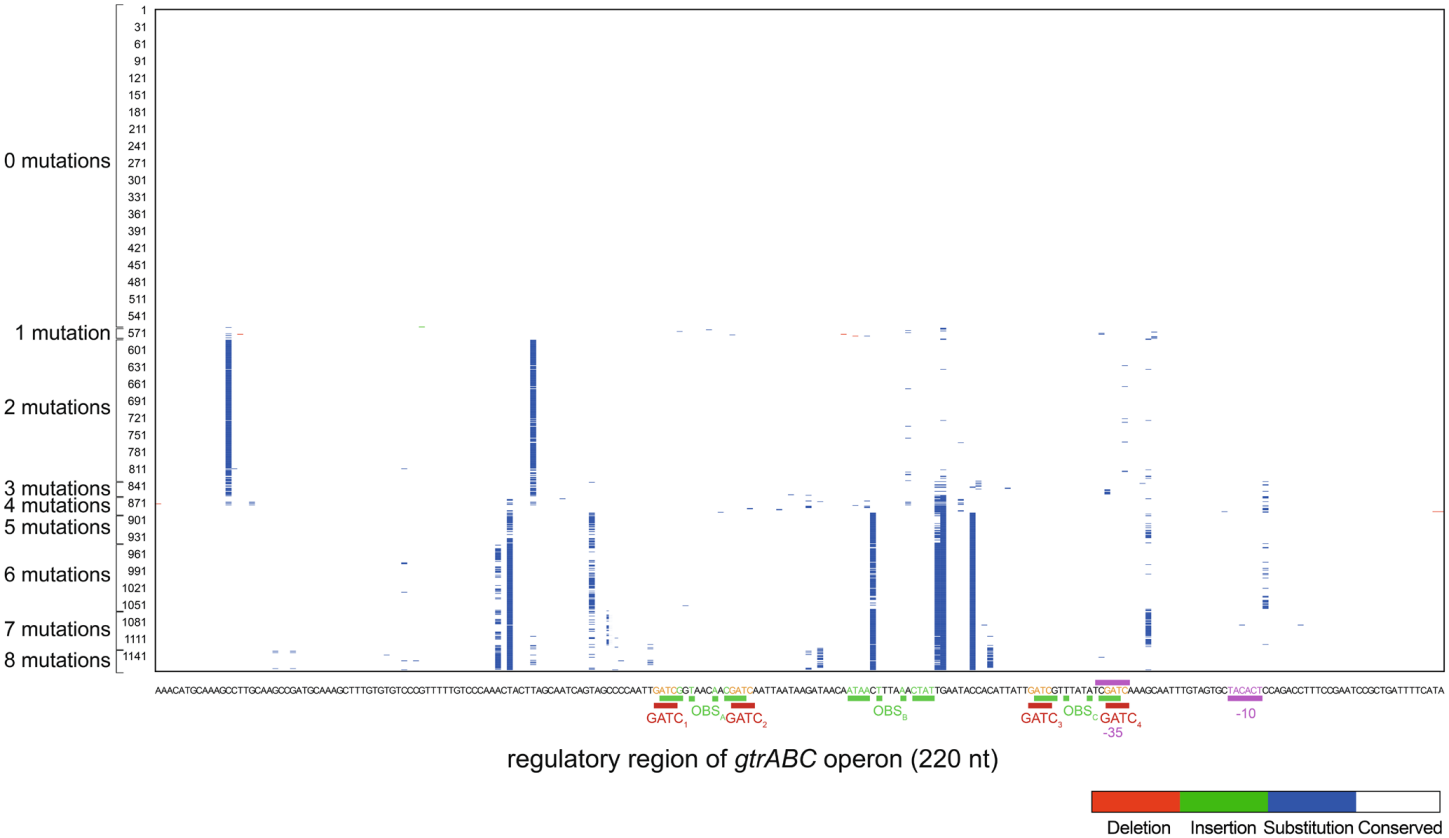
Intra‐subspecies mutational distribution in the regulatory region of the *gtrABC* operon in *Salmonella enterica* subsp. *enterica*. Localisation and distribution of mutations within the 220 nucleotides upstream of the *gtrA* start codon across *S. enterica* subsp. *enterica* genomes carrying between 1 and 8 mutations. Each row represents a genome, and each column corresponds to a nucleotide position. The nucleotide sequence of the ATCC 14028 strain used as reference is shown below the heatmap, with key regulatory elements such as GATC sites, OxyR binding sites (OBSs) and the −35 and −10 promoter boxes highlighted in colours (Broadbent et al. 2010). Nucleotides corresponding to conserved positions in the OBS consensus sequence are highlighted in green. Mutations are categorised as deletions (red), insertions (green) and substitutions (blue), while conserved nucleotides are represented in white.

Additionally, we conducted an in‐depth analysis of the nucleotide sequence variability across the entire *opvAB* regulatory region to better understand its distribution and potential functional implications. We classified *S. enterica* subsp. *enterica* genomes based on the number of sequence variations relative to the reference genome, confirming the region's high variability. Notably, only 26.7% of genomes showed complete identity with the reference sequence (Table 1, Figure 4). Polymorphism mapping revealed that sequence changes tend to occur at specific, recurrent positions rather than being randomly distributed (Figure 4a). This non‐random pattern suggests a structured and potentially functional form of variability that may confer adaptive advantages. Importantly, similar trends in high sequence variability and non‐random distribution were also observed in the regulatory region of the *gtrABC* operon (Figure 5), further reinforcing these observations.

**TABLE 1:** Summary of sequences used to mutation localisation in *opvAB* and *gtrABC* regulatory region of *Salmonella enterica* subsp. *enterica*. Number of sequences retrieved from BLASTn using the 220 nucleotides upstream of the *opvA* and *gtrA* start codons. Sequences were grouped according to the number of nucleotide changes (ranging from 0 to 8) relative to the reference. Identity percentages corresponding to each group are also indicated.

No. Mutations	Percentage of identity (PI)	No. sequences from BLASTn (*opvAB*)	% sequences from BLASTn (*opvAB*)	No. sequences from BLASTn (*gtrABC*)	% sequences from BLASTn (*gtrABC*)
0	100	454	26.7	560	48
1	99.55	325	19.1	21	1.8
2	99.09	387	22.8	251	21.5
3	98.64	54	3.2	32	2.7
4	98.18	269	15.8	24	2.1
5	97.73	76	4.5	54	4.6
6	97.27	88	5.2	118	10.1
7	96.82	41	2.4	66	5.7
8	96.36	6	0.3	40	3.4

To gain further insight into the biological relevance of these in silico observations, we analysed *Salmonella* isolates collected from poultry farms, aiming to assess whether the regulatory variability observed in genomic databases is also present and potentially functional in field conditions where bacteria face diverse environmental pressures. We compared the *opvAB* operon sequence from eight strains collected from diverse poultry farms around Salamanca (Spain), whose whole genome analysis revealed that these isolates belong to different serovars of *Salmonella enterica* subsp. *enterica* (Table 2). This *opvAB* operon comparative analysis showed that three of them (SV10243, SV10251 and SV6818) have identical sequences to that of ATCC 14028 (Figure 4b), whereas the remaining five (SV10191, SV10192, SV10193, SV10194 and SV10195) showed minor changes in the regulatory region, between 2 and 5 substitutions (0.9%–2.3%), none of them affecting the GATC sites. OBS_B_, OBS_C_ and OBS_D_ were fully conserved in this strain set. Substitutions were found in OBS_A_ in sequences from isolates SV10193 and SV10194 (Figure 4b). This variation in OBS_A_ was also observed in public genome database (Figures 4a, 2c and 3), supporting the recurrence of changes at specific sites.

**TABLE 2:** Strains of *Salmonella enterica* used in this study.

Strain name	Genotype	References
ATCC 14028	Wild type	ATCC
SV10191	Environmental isolate number 1 ( *Salmonella enterica* subsp. *enterica* serovar Enteritidis)	This study BioProject: PRJEB56392
SV10243	Environmental isolate number 2	This study
SV10192	Environmental isolate number 3 ( *S. enterica* serovar Kentucky)	This study BioProject: PRJEB56393
SV10193	Environmental isolate number 4 ( *S. enterica* serovar Bredeney)	This study BioProject: PRJEB56394
SV10194	Environmental isolate number 5 ( *S. enterica* serovar Bredeney)	This study BioProject: PRJEB56395
SV10251	Environmental isolate number 6	This study
SV10195	Environmental isolate number 7 ( *S. enterica* serovar Enteritidis)	This study BioProject: PRJEB56396
SV6818	Environmental isolate number 8	This study
SV10095	SV10191 *opvB::gfp*	This study
SV10096	SV10192 *opvB::gfp*	This study
SV10097	SV10193 *opvB::gfp*	This study
SV10098	SV10194 *opvB::gfp*	This study
SV10099	SV10195 *opvB::gfp*	This study
SV6727	14028 *opvB::gfp*	Cota, Bunk, et al. (2015); Cota, Sánchez‐Romero, et al. (2015)
SV8701	14028 OBS_A_ ^a^ *opvB::gfp*	Cota, Bunk, et al. (2015); Cota, Sánchez‐Romero, et al. (2015)
SV8695	14028 OBS_B_ ^a^ *opvB::gfp*	Cota, Bunk, et al. (2015); Cota, Sánchez‐Romero, et al. (2015)
SV8702	14028 OBS_C_ ^a^ *opvB::gfp*	Cota, Bunk, et al. (2015); Cota, Sánchez‐Romero, et al. (2015)
SV8696	14028 OBS_D_ ^a^ *opvB::gfp*	Cota, Bunk, et al. (2015); Cota, Sánchez‐Romero, et al. (2015)

^a^ OxyR binding site with point mutations.

Overall sequence analysis from public repositories (Figure 4a) and isolates obtained from poultry farms (Figure 4b) revealed the presence of specific positions within the *opvAB* regulatory region that are more prone to mutation—so‐called ‘hot spots’—while key regulatory elements such as the −35 and −10 promoter regions and GATC sites remained highly conserved. Interestingly, OBS_B_ and OBS_D_ were highly conserved (Figures 2 and 3), but OBS_A_ and OBS_C_ displayed greater variability. The differential conservation observed—especially at the OBS_A_—suggests that variability at these positions may play a role in fine‐tuning *opvAB* expression. According to the tentative models published in (Broadbent et al. 2010; Cota, Bunk, et al. 2015; Cota, Sánchez‐Romero, et al. 2015), OxyR dimers may bind independently the OBS_A_ and OBS_C_ to favour the predominant OFF state. Thus, sequence heterogeneity at these sites could influence the stability or affinity of OxyR binding, potentially shifting the balance between ON and OFF states and contributing to strain‐specific differences in *opvAB* regulation. Conversely, strong conservation of the GATC sites and core promoter elements (−35 and −10) likely reflects their essential roles in maintaining basic regulatory function. This structured variability may be functional, allowing *Salmonella* to flexibly respond to environmental changes while preserving critical regulatory mechanisms, and may constitute an additional layer of regulatory evolution in this species.

### 2.4 Functional Implications of the Variability on the Regulatory Sequence of the *opvAB* Epigenetic System

**FIGURE 6:**
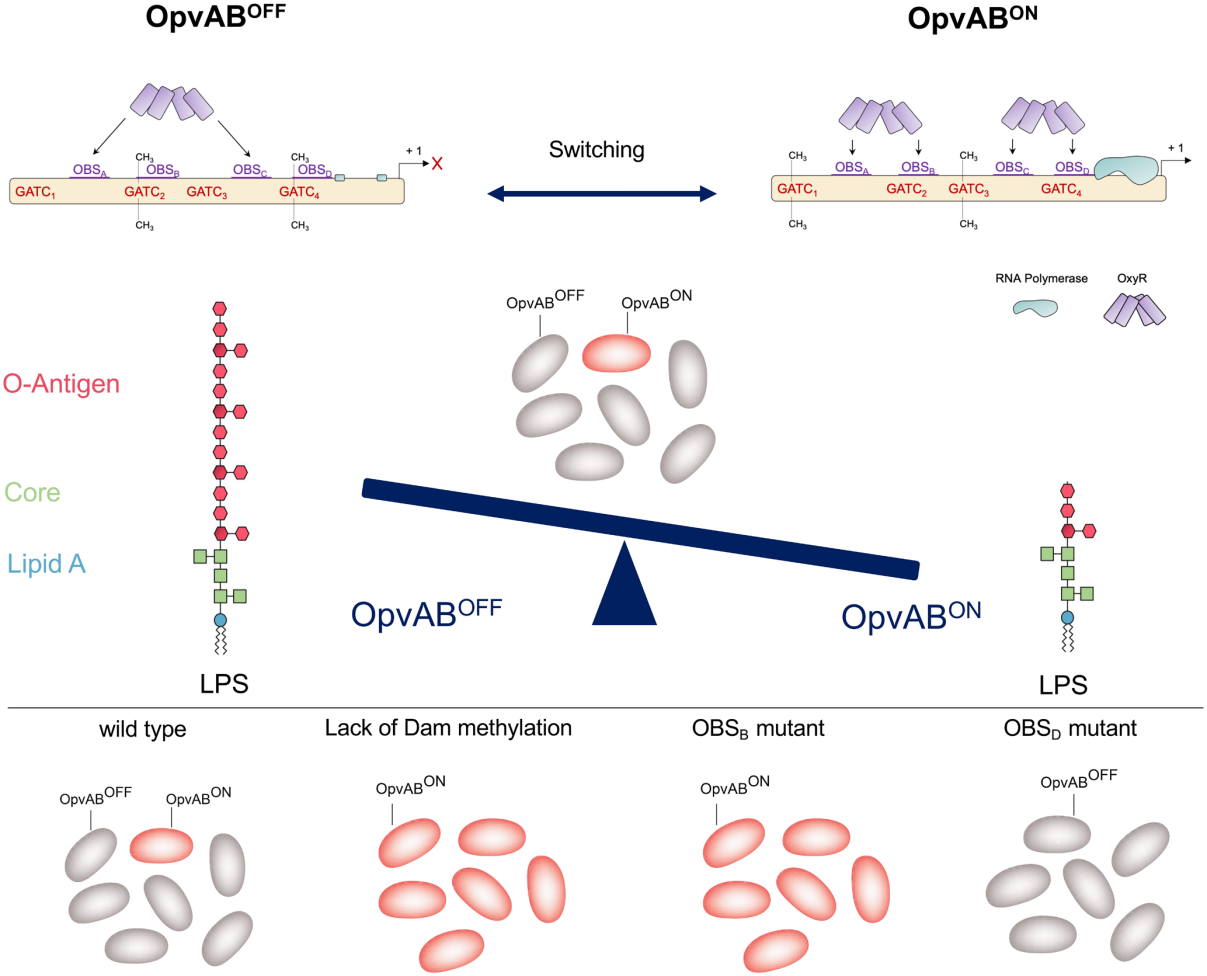
Schematic diagram of the *opvAB* regulatory region, with the GATC sites and the OxyR binding sites (OBS) outlined. The *opvAB* operon encodes proteins that alter the O‐antigen chain length of the LPS. The expression of *opvAB* undergoes phase variation and produces two subpopulations: The majority of the population is in the OFF state; these cells do not express the O‐antigen and present long O‐antigens. In contrast, a minority of the population express the *opvAB* genes (ON state). When these genes are expressed, the O‐antigen length is shortened, and these cells present a short O‐antigen (middle panel). *opvAB* transcription is controlled by the LysR‐type transcription factor OxyR and by Dam methylation. In the regulatory region of *opvAB*, there are four OBS and four GATC sites susceptible to be methylated by Dam (upper panel). OFF and ON subpopulations present opposite methylation patterns in the regulatory region of *opvAB*. Mutations in Dam, OBS_B_ and OBS_D_ abolish the phase variation (lower panel), leading to populations locked in different *opvAB* activation states.

The *opvAB* operon generates a bacterial lineage with standard LPS structure (OpvAB^OFF^) and a lineage with shorter O‐antigen chains (OpvAB^ON^) (Figure 6). OpvAB^ON^ and OpvAB^OFF^ cell lineages exhibit mutually exclusive DNA methylation patterns at the *opvAB* regulatory region, specifically at GATC_1–4_ sites, established through OxyR‐dependent binding (Cota, Bunk, et al. 2015; Cota, Sánchez‐Romero, et al. 2015).

Expression analysis of an *opvAB*::*gfp* transcriptional fusion, monitored by flow cytometry in the strain *S. enterica subsp. enterica* serovar Typhimurium ATCC 14028 cultured under standard laboratory conditions, detect a major OpvAB^OFF^ subpopulation (~99.8%) and a minor OpvAB^ON^ subpopulation (~0.2%) (Cota, Bunk, et al. 2015; Cota, Sánchez‐Romero, et al. 2015). According to Cota, Bunk, et al. (2015); Cota, Sánchez‐Romero, et al. (2015), *dam* mutant, mutated GATC sites and mutations in OBS_B_ and OBS_D_ of the *opvAB* regulatory region abolish phase variation and lock the system in the ON (*dam* mutant or mutated GATCs or OBS_B_) or OFF (mutations in OBS_D_) state. Figure 6 provides a schematic summary of these findings, illustrating how specific mutations in regulatory elements shift the balance between ON and OFF states and thereby explain the observed population distribution.

**FIGURE 7:**
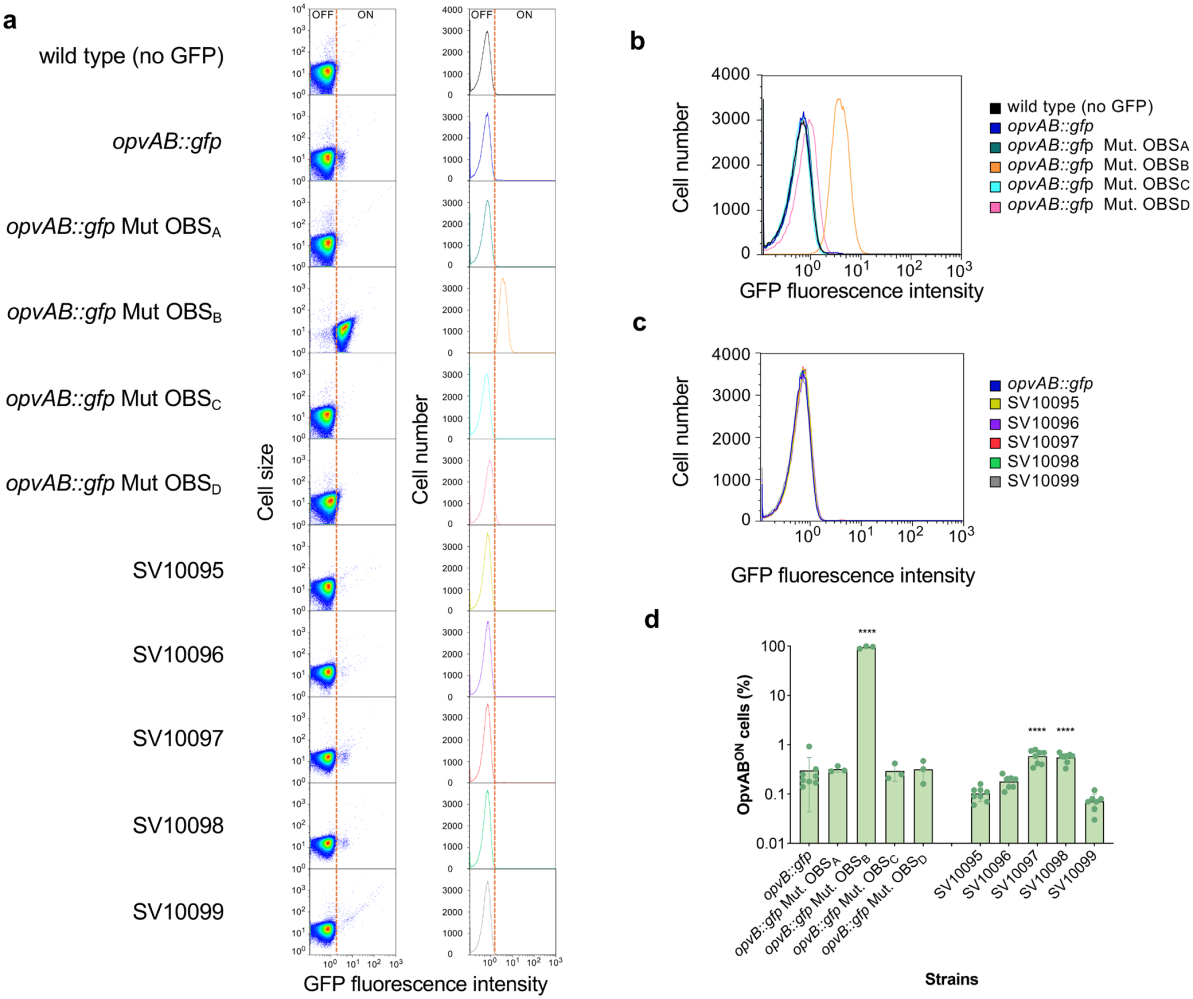
Impact of regulatory sequence variability in the *opvAB* epigenetic system on phase variation. (a) Flow cytometry analysis of *opvAB::gfp* expression in 14028 (wild‐type strain), OBS_A_, OBS_B_, OBS_C_ and OBS_D_ mutants and isolates obtained from poultry farms containing *gfp* transcriptional fusions. Dot plots and histograms are represented. GFP‐ON and GFP‐OFF populations were defined based on fluorescence intensity histograms. The gating threshold was established using a GFP‐negative control, with cells exceeding the upper limit of basal fluorescence (99th percentile) classified as GFP‐ON. Analyses were performed in triplicate and eight times for OBSs mutants and environmental strains, respectively, and a representative experiment is shown. (b) Distribution of GFP fluorescence intensity in *Salmonella* strains carrying different *opvAB* variants. Strains harbouring *opvAB*::*gfp* with the wild‐type OxyR binding site or with mutations in OxyR binding sites (Mut. OBS_A_, Mut. OBS_B_, Mut. OBS_C_ and Mut. OBS_D_) are shown. Histograms represent single‐cell fluorescence measured by flow cytometry. (c) Distribution of GFP fluorescence intensity in *Salmonella* isolates obtained from poultry farms carrying *gfp* transcriptional fusions. The reference strain *opvAB*::*gfp* and isolates SV10095, SV10096, SV10097, SV10098 and SV10099 are shown. Histograms represent single‐cell fluorescence measured by flow cytometry. (d) Proportion of cells expressing *opvAB* in different OxyR binding site mutants and environmental strains monitored by flow cytometry. Data from eight independent flow cytometry trials are shown. All comparisons were made against the reference strain 14028 *opvB::gfp*. Statistical indications: ****p < 0.0001. Not significantly differences are not shown.

Based on the existing sequence variation in the *opvAB* OxyR binding sites across genomes, we next investigated the effect of promoter sequence variation on gene expression and on the formation of ON and OFF cell lineages. First, we employed *S. enterica* 14028 and derivative strains previously engineered via site‐directed mutagenesis to alter specific OxyR binding sites, and analysed how the degree of nucleotide identity with the OxyR consensus binding sequence contributes to *opvAB* phase variation (Cota, Bunk, et al. 2015; Cota, Sánchez‐Romero, et al. 2015). Of the four OxyR binding sites present in the *opvAB* regulatory region of this strain, OBS_A_ and OBS_C_ are an absolute match (10 out of 10 nucleotides) to the consensus sequences defined for OxyR binding (Figure S2). In contrast, OBS_B_ and OBS_D_ share only 8 and 7 out of 10 nucleotides with the consensus sequence, respectively (Figure S2) (Storz et al. 1990; Toledano et al. 1994). The following mutations were introduced to generate imperfect matches in these OxyR binding sites: one nucleotide change was introduced in OBS_A_ and OBS_C_, and one and two in OBS_B_ and OBS_D_, respectively, causing mutated versions which share 9 out of 10 nucleotides with the consensus sequence in all cases. Importantly, consensus sequences were avoided in OBS_B_ and OBS_D_ sites because GATC_2_ and GATC_4_, Dam methylation motifs, are embedded within them and it would inevitably affect methylation (Figure S2). To evaluate the impact of these mutations, we constructed a panel of chromosomal *opvAB*::*gfp* transcriptional fusions in the ATCC 14028 genome and analysed gene expression at the single‐cell level using flow cytometry, enabling us to distinguish and quantify bacterial subpopulations in the ON and OFF states (Figure 7a,b,d). Due to the low frequency of the ON subpopulation, both dot plots and histograms (Figure 7a) are presented to ensure a clear and accurate representation of these events. Mutations in OBS_B_ or OBS_D_ abolished phase variation, resulting in homogenous populations of either OpvAB^ON^ or OpvAB^OFF^ cells, respectively. In contrast, mutations in OBS_A_ or OBS_C_ caused minor variations in the proportion of ON cells, without eliminating bimodal expression.

Secondly, to complement our genomic analyses, we returned to the set of poultry farm isolates (Table 2) to experimentally evaluate the functional consequences of their regulatory variants. To this end, we constructed *gfp* transcriptional fusions, measured *opvAB* expression by flow cytometry (Figure 7a,c) and quantified the distribution of ON and OFF subpopulations (Figure 7d). The expression patterns were consistent with our in silico observations: OBS_A_ mutations in strains SV10097 and SV10098 resulted in a slight but significant increase in the OpvAB^ON^ subpopulation (*p* < 0.0001), whereas strains SV10095, SV10096 and SV10099, which do not carry mutations in this region, did not show this effect. These findings highlight that while OBS_B_ and OBS_D_ are indispensable for maintaining phase variation, OBS_A_—and likely OBS_C_—act as modulators that fine‐tune the distribution between ON and OFF states, adding flexibility to the regulatory system.

It is interesting to note that strains SV10193 and SV10194 both belong to *Salmonella enterica* serovar Bredeney (Table 2), which is frequently linked to poultry farms (Arvanitidou et al. 1998; Limawongpranee et al. 1999; Iannetti et al. 2020), and exhibited a higher proportion of the OpvAB^ON^ subpopulation when their *opvAB* gene was tagged with the GFP reporter (strains SV10097 and SV10098, shown in Figure 7). Poultry farm environments represent an important reservoir of *Salmonella* phages due to the high bacterial load and specific ecological conditions (Sevilla‐Navarro et al. 2020). In this context, the slightly elevated proportion of the OpvAB^ON^ (phage‐resistant phenotype) subpopulation observed in these Bredeney strains, likely driven by sequence variation in the OBS_A_ region, may confer an adaptive advantage to persist in environments where bacteriophage exposure is frequent.

These results suggest that changes at specific positions within the regulatory region can modulate the frequency of gene expression while preserving basic promoter functionality. A larger or smaller OpvAB^ON^ subpopulation may provide selective advantages under fluctuating environmental conditions (Cota, Bunk, et al. 2015; Cota, Sánchez‐Romero, et al. 2015; Fernández‐Fernández et al. 2024). However, abolishing phase variation as observed in OBS_B_ and OBS_D_ mutants, eliminates such adaptive flexibility and could be lethal, all supporting the importance of these regulatory elements to maintaining the OFF/ON balance. In contrast, the observed tolerance to mutations in OBS_A_ and OBS_C_ may reflect an evolutionary strategy for probabilistic (i.e., stochastic) gene expression control, generating phenotypic heterogeneity within the population. Such variability can enhance the chances of survival under fluctuating or stressful environmental conditions.

Given the functional role of *opvAB* in modulating surface antigen expression, its regulatory plasticity likely confers adaptive advantages under conditions of intense selective pressure. This is particularly relevant in environmental isolates from poultry farms, where high bacterial densities and intense microbial competition create a dynamic ecological niche. As demonstrated by Cota, Bunk, et al. (2015); Cota, Sánchez‐Romero, et al. (2015), phase variation mediated by *opvAB* enables reversible switching between phenotypic states that affect LPS structure, thereby influencing phage susceptibility, immune evasion and host colonisation. Such phenotypic heterogeneity may be crucial for survival in agricultural environments, where frequent exposure to antimicrobials, bacteriophages and host immune responses demands flexible and rapid adaptation. The evolution of phase‐variable systems like *opvAB* thus appears tightly linked to ecological pressures that favour fine‐tuned control of surface structures. The presence of structured promoter variation across diverse *Salmonella* subspecies and niche‐adapted serovars—including both broad‐host and host‐restricted lineages—further supports its evolutionary significance. We propose that this variation complements classical genetic and epigenetic regulation by enabling dynamic, fine‐scale control of gene expression without disrupting core promoter function.

Future research will focus on characterising the specific environmental cues that select for different *opvAB* regulatory variants and investigating the prevalence of these variants in diverse ecological niches. The insights gained from this study contribute to our understanding of bacterial adaptation strategies and the evolutionary dynamics of horizontally acquired regulatory systems.

## 3 Methods

### 3.1 Bacterial Strains

The strains of *Salmonella enterica* used in this study are listed in Table 2. Unless otherwise noted, *Salmonella enterica* strains belong to serovar Typhimurium and are derived from the mouse‐virulent strain ATCC 14028. Environmental *Salmonella* isolates SV10191, SV10192, SV10193, SV10194 and SV10195 were sequenced as part of this work. Their corresponding serovars and associated BioProject accession numbers are also detailed in Table 2.

### 3.2 Media and Growth Conditions

Bertani's lysogeny broth (LB) was used as standard growth medium and contained tryptone (10 g/L), yeast extract (5 g/L) and NaCl (5 g/L). Solid media contained agar at 1.5% final concentration. Cultures were grown at 37°C, or at 30°C when strains carried the thermosensitive pKD46 plasmid. When required, ampicillin and chloramphenicol were used at 100 μg/mL and 20 μg/mL, respectively. Aeration of liquid cultures was obtained by shaking at 200 rpm in a Multitron shaker (Infors HT).

### 3.3 Bacterial Strains Construction

For construction of *opvB::gfp* transcriptional fusions at the 3′ end of the *opvB* coding sequence, a DNA fragment containing the promoterless green fluorescent protein gene (GFP) and the chloramphenicol resistance cassette was amplified by PCR from plasmid pZEP07 (Hautefort et al. 2003) using oligonucleotides STM2208stop‐GFP‐5 and STM2208stop‐GFP‐3 (Table 3). After PCR product purification using the commercial Wizard SV Gel and PCR Clean‐Up System Kit (Promega), the construct was integrated into the chromosome of environmental *Salmonella* strains using the lambda Red recombination system (Datsenko and Wanner 2000). Verification of the constructs was carried out using setB‐For and STM2208stop‐GFP‐5 oligonucleotides (Table 3).

**TABLE 3:** Oligonucleotides used in this study.

Oligonucleotide name	Sequence (5′‐3′)
*setB*‐For	CTGCCTGGCGCGTATTAAAGAT
*yeiR*‐Rev	GCTGGAACTCGAACAGAAAACCTTACC
STM2208‐E1	AATATACGTTCGCAGCCAGG
*opvB*‐Rev	TCATGCGCGGTCATTCATTG
STM2209‐E2	TTGTATCATGCTGCACGCTC
*yeiW*‐Rev	GCTGGAACTCGAACAGAAAACCTTACC
STM2208stop‐GFP‐3	ACTTTTACTCTTCGACACATTTCAGCGCAGAGTTTATCTCTGCGCAATGTTTATCACTTATTCAGGCGTA
STM2208stop‐GFP‐5	CGCTAACAGAATATCGTATTGAGAAAAAGACAATGAATGACCGCGCATGA

### 3.4 Sequence Diversity of Surface‐Associated and Housekeeping Genes

Genome sequence of *Salmonella enterica* subsp. *enterica* serovar Typhimurium strain 14028S (GenBank: CP001363.1; Assembly: GCA_000022165.1) was obtained from the National Center for Biotechnology Information (NCBI) and used as reference. The nucleotide sequences corresponding to the open reading frames (ORFs) and the 220 nucleotides upstream of the start codons were retrieved for all the genes of *Salmonella* using a custom script. The Basic Local Alignment Search Tool (BLASTn) (https://blast.ncbi.nlm.nih.gov/) was used to search these sequences against all complete and fully annotated genomes of the genus *Salmonella* (taxid: 590), using the NCBI non‐redundant nucleotide (nr/Refseq) database (accessed December 2025). A total of 1908 fully annotated *Salmonella* genomes were analysed, as the dataset was restricted to genomes annotated by the NCBI. To ensure data quality, only the highest‐scoring hit per genome was retained, considering only alignments with a minimum query coverage of 50%. The resulting sequences were downloaded and sorted by their percentage of identity (PI) to the reference sequence in 1% intervals. Genes were subsequently classified into two functional groups (surface‐associated genes and housekeeping genes) based on their Gene Ontology (GO) Biological Process (GO_PROCESS) annotations (Table S1).

Surface‐associated genes were identified as those annotated with following GO terms: transmembrane transport, ion transmembrane transport, amino acid transmembrane transport, carbohydrate transport, peptide/oligopeptide transport, nucleoside/nucleobase transport and metal ion transport, as well as xenobiotic transport, siderophore transport, phosphate/sulphate transmembrane transport and phosphoenolpyruvate‐dependent sugar phosphotransferase system. This category also integrated the cell wall organisation and biogenesis, and peptidoglycan biosynthetic/catabolic/metabolic process as well as LPS biosynthetic process, lipid A biosynthetic process, O‐antigen, biosynthetic process and enterobacterial common antigen biosynthetic process. Furthermore, terms related to Gram‐negative‐bacterium‐type cell outer membrane assembly, lipoprotein biosynthetic process and transport, membrane assembly, protein secretion, protein export, protein transport by the Sec complex and SRP‐dependent cotranslational protein targeting to membrane were included. Type I, II and VI secretion systems, signal peptide processing, protein localisation to the outer membrane, bacterial‐type flagellum assembly, flagellum‐dependent cell motility, chemotaxis, pilus assembly and type IV pilus‐dependent motility were also categorised as surface‐associated, alongside cell adhesion, biofilm formation, phosphorelay signal transduction systems, signal transduction and two‐component system–related terms.

Housekeeping genes were defined as those involved in the translational machinery, specifically focusing on GO terms such as translation, translational initiation, elongation and termination, ribosome biogenesis, ribosomal subunit assembly, tRNA aminoacylation, tRNA processing and modification, and rRNA processing or methylation.

### 3.5 Correlation Between Coding (CDS) and Regulatory Sequences (RS)

The coding sequences of the reference genome *Salmonella enterica* subsp. *enterica* serovar Typhimurium strain 14028S (GenBank: CP001363.1; Assembly: GCA_000022165.1) were selected using a custom script. The corresponding regulatory sequences were extracted and defined as the 220 nucleotides (nt) upstream of the start codon. Using this set of sequences, BLASTn searches were performed against all complete *Salmonella* genomes available in the NCBI database. For each genome, the highest‐quality hit was selected, considering only alignments with a minimum coverage of 50%. All resulting alignments were downloaded, and their percentage of identity (PI) was recorded. Based on these data, the mean percentage identity of the alignments obtained for both coding sequences and regulatory sequences was calculated for each gene. This approach yielded a final average value that reflects the degree of conservation of the coding and regulatory sequences of each individual gene across the *Salmonella* genus. Subsequently, the relationship between the nucleotide conservation in coding and regulatory regions was analysed using a custom script. The Pearson correlation coefficient was calculated, and a scatter plot was generated using R software (version 2023.09.1) within the RStudio environment, comparing the mean percentage identity of coding sequences (CDSs) and that of regulatory sequences (RSs) across the 4743 genes comprising the *Salmonella* genome.

### 3.6 GC Content Analysis

The nucleotide sequence of *opvAB* and its surrounding genes (~1.5 Kb upstream and downstream of *opvAB*) was collected from the National Center for Biotechnology Information (NCBI) database. This sequence belonged to the strain *Salmonella enterica* serovar Typhimurium ATCC 14028 (GenBank accession number CP001363.1). SnapGene software version 8.1 (Insightful Science) was used to plot the GC content with a sliding window of 25 nucleotides.

### 3.7 Distribution of Mutations in the Regulatory Elements of the *opvAB* Across *Salmonella enterica* Subspecies

To assess the diversity of regulatory elements of *opvAB* among different *Salmonella enterica* subspecies, a BLASTn search was conducted using the 220 nucleotides upstream of the *opvA* start codon. The search was performed against all complete and fully annotated genomes of the genus *Salmonella* (taxid: 590) available in the NCBI nr/RefSeq database. Retrieved sequences were classified into four *Salmonella enterica* subspecies: *enterica* (*n* = 1717), *arizonae* (*n* = 14), *diarizonae* (*n* = 20) and *salamae* (*n* = 18). Sequences were aligned to the reference genome *Salmonella enterica* serovar Typhimurium ATCC 14028 (GenBank accession number CP001363.1) using MUSCLE implemented in the software Molecular Evolutionary Genetics Analysis (MEGA) X (Kumar et al. 2018). Seven sequences from subsp. *enterica* were excluded due to insertions or deletions that interfered with accurate alignment of regulatory motifs. Consequently, the final dataset included 1710 sequences of the subsp. *enterica*. Mutational patterns in key regulatory elements, including the −35 and −10 promoter boxes, four GATC sites and four OxyR binding sites (OBSs), were visualised as a heatmap using GraphPad Prism version 9.

### 3.8 Analysis of Mutation Distribution Within the *opvAB* and *gtrABC* Regulatory Region in *Salmonella enterica* Subsp. *enterica* Genomes

To examine the distribution of mutations within the regulatory region of the *opvAB* and *gtrABC* operons, the sequences encompassing the 220 nucleotides upstream of the *opvA* and *gtrA* start codons from the genome of *Salmonella enterica* subsp. *enterica* serovar Typhimurium strain 14028S (GenBank accession number CP001363.1) were searched in all complete and fully annotated genomes of *Salmonella enterica* subsp. *enterica* available in the NCBI database. Searches were performed using the BLASTn algorithm, and hits were filtered to retain only sequences with 100% query coverage. Sequences carrying between 0 and 8 mutations relative to the reference were retrieved for further analysis (Table 1). These sequences were aligned using the MUSCLE algorithm implemented in MEGA (Kumar et al. 2018) and examined to determine the precise positions and types of mutations within the 220‐nucleotide regulatory region.

### 3.9 Sequence Variation in the Regulatory Region of the *opvAB* Operon in Environmental Strains of *Salmonella enterica*

To analyse sequence variation in the regulatory region of *opvAB* operon from environmental *Salmonella* strains, nucleotide amplification by PCR was performed using oligonucleotides *setB*_For and *yeiW*_Rev (Table 3). PCR products were then cleaned using the commercial Wizard SV Gel and PCR Clean‐Up System Kit (Promega) and sequenced by Stabvida using the oligonucleotides previously employed for the PCR. Sequences were aligned using the MUSCLE algorithm implemented in MEGA X in order to identify nucleotide variations.

### 3.10 Whole Genome DNA Sequencing and Analysis

Bacterial genome sequencing and bioinformatic analysis were performed by AllGenetics & Biology SL. Genomic DNA was isolated with the Easy‐DNA kit (Invitrogen), following the manufacturer's instructions. Quantity and quality of genomic DNA were measured by Qubit High Sensitivity dsDNA Assay (Thermo Fisher Scientific) and Nanodrop ND‐1000 spectrophotometer (Thermo Fisher Scientific). Genomic libraries were prepared using the Nextera XT Library Prep kit (Illumina) and its purification was performed by Mag‐Bind RXNPure Plus magnetic beads (Omega Bioteck). Libraries were pooled in equimolar amounts and sequenced in the platform NovaSeq PE150 run (Illumina), with a total input of 15 gigabases. Sequencing yielded between 3,351,908 and 5,784,360 paired‐end reads. The software used to align the reads to their reference genomes was Snippy (https://github.com/tseemann/snippy). The strain ATCC 14028 was used as reference genome. Single Nucleotide Polymorphisms (SNPs), insertions and deletions (indels) were localised in *Salmonella* genome by using the *Salmonella enterica* subsp. *enterica* serovar Typhimurium ATCC 14028 genome deposited in GenBank with the accession number NZ_CP034479.1.

### 3.11 Flow Cytometry

Flow cytometry was used to monitor expression of the *opvAB::gfp* transcriptional fusions. Bacterial cultures were grown at 37°C in LB until exponential phase (O.D._600nm_ = 0.4). Cells were then washed and re‐suspended in phosphate‐buffered saline (PBS) for fluorescence measurement. Data acquisition was performed using a Cytomics FC500‐MPL cytometer (Beckman Coulter, Brea, CA). All data were collected for 100,000 events per sample and analysed with FlowJo version 8.7 software. Fluorescence values were compared with the data from the reporter less control strain, thus yielding the fraction of ON and OFF cells. Data are represented either by both dot plot (side scatter [cell size] vs GFP fluorescence intensity) and histogram (cell number vs GFP fluorescence intensity). GFP^ON^ and GFP^OFF^ populations were defined based on fluorescence intensity histograms. The gating threshold was established using a GFP‐negative control, with cells exceeding the upper limit of basal fluorescence (99th percentile) classified as GFP^ON^. The percentage of ON cells is plotted in a bar chart by GraphPad Prism version 9 software.

### 3.12 Statistical Analysis

Statistical analysis was performed using GraphPad Prism version 9. One‐way ANOVA was performed to assess differences among groups, and multiple comparisons were corrected using Dunnett's post hoc test, with the *Salmonella* 14028 *opvB::gfp* strain as control. Experiments were conducted at least three times. A *p* value of < 0.05 was considered statistically significant.

## Author Contributions

R.F.F. contributed to conceptualisation, formal analysis, investigation, methodology, writing – original draft, review and editing; G.G. and F.P. contributed to formal analysis, investigation and methodology; C.R.B. contributed to conceptualisation and writing – original draft, review and editing; J.G. contributed to writing – review and editing; and M.A.S.‐R. contributed to conceptualisation, formal analysis, investigation, methodology, funding acquisition, supervision, writing – original draft, review and editing.

## Funding

This work was supported by Ministerio de Ciencia, Innovación y Universidades, PID2023‐151613OB‐I00, CNS2022‐135641, PID2021‐125947OB‐I00, PID2024‐155918OB‐I00, PID2021‐127245OB‐I00, PID2024‐160046OB.

## Conflicts of Interest

The authors declare no conflicts of interest.

## Supporting information


**Table S1:** mbt270312‐sup‐0001‐DataS1.zip.
**Figure S1:** Genome‐wide correlation analysis between the percent identity (PI) of the coding sequences (CDS) and their corresponding regulatory sequences (RS) in *Salmonella*. The scatter plot displays the relationship between the average percent identity (PI) of regulatory sequences (RS, *x*‐axis) and their respective coding sequences (CDS, *y*‐axis) for the 4743 genes comprising the *Salmonella* genome. Each data point represents a single gene and shows the mean PI calculated from genomic alignments to the reference sequence. The Pearson correlation coefficient (*R* = 0.311, *p* < 2.22 × 10^−16^) indicates a statistically significant but weak positive relationship, suggesting that evolutionary pressures act with a substantial degree of independence on these two functional regions. A magnified view of the region spanning 95%–100% PI is shown and highlighted by a red dashed box.
**Figure S2:** OxyR binding sites in the *opvAB* regulatory region. The *opvAB* OxyR binding sites A, B, C and D of a *S.* *enterica* 14028 wild‐type strain, mutant derivatives and five environmental strains are shown along with the consensus OxyR binding sequence (Storz et al. 1990; Toledano et al. 1994). GATC sequences are underlined. Variations in OxyR binding sites compared with the consensus sequence are marked in bold. ‘N’ denotes any nucleotide. Nucleotides that are not part of the consensus sequence are shown in black, whereas consensus nucleotides are colour‐coded as follows: A in red, T in blue, G in green and C in purple.
**Table S1:** Functional categorization of *Salmonella* genes. Genes were assigned to surface‐associated or translation‐related categories according to their Gene Ontology (GO) Biological Process annotations. The number of genes associated with each GO term is indicated.

## Data Availability

Genome sequencing data generated in this study are available at the European Nucleotide Archive (ENA) under BioProject accession numbers PRJEB56392, PRJEB56393, PRJEB56394, PRJEB56395 and PRJEB56396. The datasets generated and analysed during this study are available in the *idUS* repository, (University of Seville) at https://hdl.handle.net/11441/177104, reference number https://doi.org/10.12795/11441/177104.
